# Epigallocatechin-3-gallate protects bovine ruminal epithelial cells against lipopolysaccharide-induced inflammatory damage by activating autophagy

**DOI:** 10.1186/s40104-024-01066-9

**Published:** 2024-08-09

**Authors:** Wanli Zhao, Taiyu Shen, Bichen Zhao, Moli Li, Zhaoju Deng, Yihui Huo, Ben Aernouts, Juan J. Loor, Androniki Psifidi, Chuang Xu

**Affiliations:** 1 National Key Laboratory of Veterinary Public Health and Safety, College of Veterinary Medicine, China Agricultural University, 2 Yuanmingyuan West Road, Beijing, 100193 China; 2 Department of Biosystems, Division of Animal and Human Health Engineering, KU Leuven University, Kleinhoefstraat 4, Geel, 2440 Belgium; 3 Department of Animal Sciences, Division of Nutritional Sciences, University of Illinois, Urbana-Champaign, Urbana, IL 61801 USA; 4 Department of Clinical Science and Services, Queen Mother Hospital for Animals, The Royal Veterinary College, North Mymms, Hawkshead Lane, Hatfield, Hertfordshire, AL9 7TA UK

**Keywords:** Bovine ruminal epithelial cell, Epigallocatechin-3-gallate, Inflammation, Subacute ruminal acidosis

## Abstract

**Background:**

Subacute ruminal acidosis (SARA) causes an increase in endotoxin, which can induce immune and inflammatory responses in the ruminal epithelium of dairy cows. In non-ruminants, epigallocatechin-3-gallate (EGCG), a major bioactive ingredient of green tea, is well-known to alleviate inflammation. Whether EGCG confers protection against SARA-induced inflammation and the underlying mechanisms are unknown.

**Results:**

In vivo, eight ruminally cannulated Holstein cows in mid-lactation were randomly assigned to either a low-concentrate (40%) diet (CON) or a high-concentrate (60%) diet (HC) for 3 weeks to induce SARA (*n* = 4). Cows with SARA had greater serum concentrations of tumor necrosis factor (TNF)-α and interleukin-6, and epithelium had histological signs of damage. In vitro, immortalized bovine ruminal epithelial cells (BREC) were treated with lipopolysaccharide (LPS) to imitate the inflammatory damage caused by SARA. Our data revealed that BREC treated with 10 µg/mL LPS for 6 h successfully induce a robust inflammatory response as indicated by increased phosphorylation of IκBα and nuclear factor kappa-B (NF-κB) p65. Pre-treatment of BREC with 50 µmol/L EGCG for 6 h before LPS challenge promoted the degradation of NLR family pyrin domain containing 3 (NLRP3) inflammasome through activation of autophagy, which further repressed activation of NF-κB pathway targeting Toll-like receptor 4 (TLR4). Analyses also revealed that the ECGG upregulated tight junction (TJ) protein expression upon incubation with LPS.

**Conclusions:**

Subacute ruminal acidosis causes ruminal epithelium injury and systemic inflammation in dairy cows. However, the anti-inflammatory effects of EGCG help preserve the integrity of the epithelial barrier through activating autophagy when BREC are exposed to LPS. Thus, EGCG could potentially serve as an effective therapeutic agent for SARA-associated inflammation.

**Graphical Abstract:**

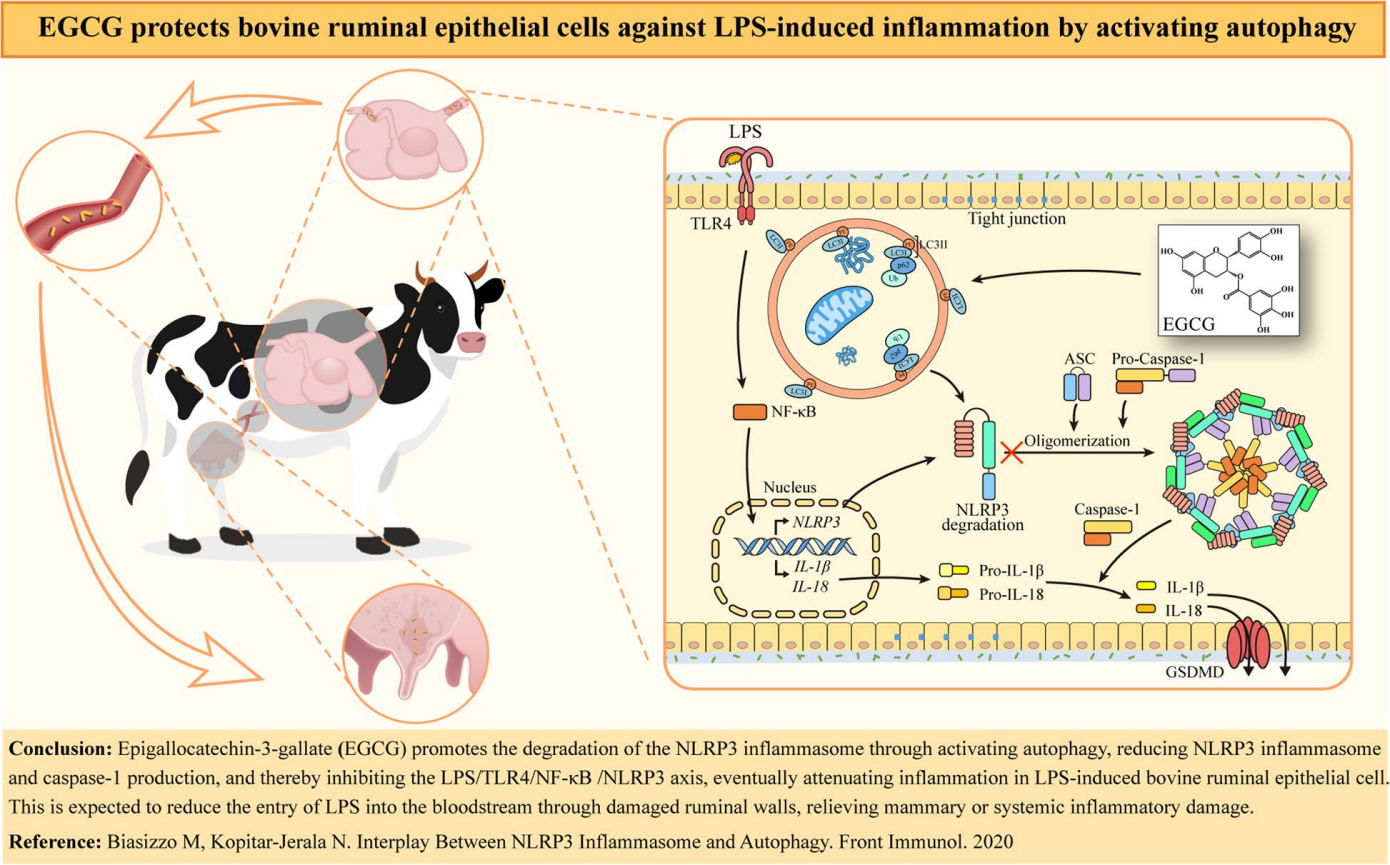

**Supplementary Information:**

The online version contains supplementary material available at 10.1186/s40104-024-01066-9.

## Background

In an effort to maximize milk production, dairy cows are fed nutrient-dense diets containing a large proportion of cereal grain, thus, increasing susceptibility to disorders such as subacute ruminal acidosis (SARA) and liver abscesses [1, 2]. Excessive intake of highly-fermentable carbohydrates affects the ruminal environment, resulting in lower pH, disturbing flora composition and abundance, and increasing levels of bioactive molecules such as lipopolysaccharides (LPS) [3, 4]. The LPS are located in the cell wall of Gram-negative bacteria, with ruminal acidosis increasing its levels 4-to-16 times compared with normal conditions [5–7]. If uncontrolled, excessive production of LPS can trigger an inflammatory response and cause inflammatory disorders that reduce productivity and cause economic losses [8].

The contact between LPS and its receptor Toll-like receptor 4 (TLR4) activates intracellular signaling through myeloid differentiation primary response protein 88 (MyD88), resulting in the activation of major translocation of nuclear factor kappa-B (NF-κB) [9]. Upon activation of the NF-κB signaling pathway, components linked to inflammasomes are upregulated. Furthermore, NLRP3 recruits the inflammasome adaptation protein apoptosis-associated speck-like protein containing a CARD (ASC), which then binds and activates caspase-1 [10]. Caspase-1 activation triggers the production of pro-inflammatory cytokines IL-1β and IL-18 [11], which damages the ruminal epithelial barrier and induces systemic inflammation.

Autophagy is an evolutionary conserved intracellular catabolic process that transfers long-lived proteins, misfolded proteins, and excess organelles to the lysosome for degradation [12–14]. Under normal circumstances, autophagy is in a basal state, while it is rapidly upregulated in response to stress factors such as nutrient and energy deficiencies, pathogen-associated molecular patterns (PAMPs) and danger-associated molecular patterns (DAMPs) [15–17]. Autophagy regulates inflammasome activation, including the NLR family pyrin domain containing 3 inflammasome (NLRP3 inflammasome), through multiple mechanisms [18]. The loss of the autophagy-related protein 16-like 1 (ATG16L1) in mouse fetal liver-derived macrophages led to an increase in caspase-1 activation and IL-1β maturation following endotoxin treatment [19]. Furthermore, increasing evidence revealed an important role of autophagy in the physiology of intestinal epithelial cells including maintenance of barrier function, suggesting that autophagy targeted therapy could be a potential treatment for diseases associated with intestinal barrier dysfunction [20, 21]. Together, available data indicate that activation of autophagy helps control the inflammatory response and maintain proper gastrointestinal barrier integrity. Whether these effects extend to the ruminal epithelial cells (BREC) is unclear.

Green tea possesses multiple physiological and pharmacological benefits, including anti-inflammatory and antioxidant effects, prevention of obesity, diabetes, cancer, and other diseases [22, 23]. The major active ingredient in green tea is epigallocatechin-3-gallate (EGCG), a potent antioxidant that can quench reactive radicals and chelate metal ions to prevent the formation of reactive oxygen species (ROS) [24]. EGCG also has anti-inflammatory effects through blocking the activation of NF-κB, activator protein-1 (AP-1), MyD88-dependent pathway, Toll-interleukin-1 receptor domain-containing adaptor inducing interferon-β (IFN-β)-dependent pathways of TLR, and the expression of cytochrome c oxidase (COX), nitric oxide (NO) synthase, and TNF-α [25, 26]. EGCG inhibited IL-6, IL-1β, and TNF-α in LPS-mediated RAW 264.7 cells [27], and induced autophagy, anti-inflammatory responses, degradation of lipid droplets in endothelial cells, and facilitated degradation of endotoxins leading to anti-inflammatory actions [28, 29]. In hepatic cells, EGCG was observed to activate Adenosine 5′-monophosphate (AMP)-activated protein kinase (AMPK), thereby advancing autophagy [30]. EGCG also diminished the effect of negative regulators of autophagy, thereby delaying apoptosis-mediated cell death and extending cell viability [31]. Whether EGCG confers protective effects against LPS-induced inflammatory responses in BREC and the underlying mechanisms are unknow.

We hypothesized that in vivo SARA has a negative effect on the BREC due to the increased release of endotoxins. As such, supplementation of EGCG in vitro would alleviate LPS-induced inflammatory and tight junction (TJ) damage by regulating autophagy. Thus, the objective of this study was to use BREC as an in vitro model when cells were incubated with EGCG and LPS. Several molecular indicators of inflammation, autophagy, and tight junction components were measured in BREC to address our hypothesis.

## Materials and methods

### Materials

EGCG (HPLC ≥ 98%) was purchased from Shanghai yuanye Bio-Technology Co., Ltd. (Shanghai, China). *Escherichia coli* LPS serotype O55:B5 was procured from Sigma-Aldrich (St. Louis, MO, USA). Chloroquine, a selective inhibitor of autophagy and TLR, was obtained from MCE (MedChemExpress, USA). A stock solution of EGCG was prepared in DMEM/F12 (Hyclone, Logan, UT, USA) medium at concentrations of 1 mmol/L. A stock solution of LPS was prepared in DMEM/F12 medium at a concentration of 1 mg/mL.

### Samples

Ruminal tissue epithelia, blood samples from cows with SARA and healthy cows were kindly provided by Dr. Shengyong Mao’s group (Nanjing Agricultural University). Data on DMI and ruminal pH of these cows were reported previously [32]. Overall, compared with the CON group, there was no significant difference in DMI due to high-concentrate feeding (23.79 vs. 22.40, *P* = 0.524). On average, ruminal pH remained at < 5.8 for 9.2 h/d due to high-concentrate feeding. Tissue was collected at slaughter and blood samples prior to sacrifice at the end of their experimental period. Serum inflammatory cytokines were detected via ELISA kits (Lengton, Shanghai, China) according to the manufacturer’s instructions. Morphology of ruminal epithelia was identified via microscopy (Olympus, Japan).

### Cell culture

Immortalized BREC were provided by Dr. Kang Zhan’s group at Yangzhou University [33]. Cells were cultured in DMEM/F12 containing 10% FBS (Gibco, Waltham, MA, USA) and 1% (v/v) penicillin/streptomycin at 37 °C in a humidified incubator with 5% CO_2_.

### Treatment of BREC

EGCG was diluted with DMEM/F12 to various concentrations (0, 10, 20, 50, 100, 125, 150, 200 and 300 µmol/L) and used to stimulate BREC for various times (2, 6 and 12 h) to test effects on the viability of BREC. LPS was dissolved in the media at specific concentrations (0, 1, 5 and 10 µg/mL) and used to stimulate BREC for 6 h to establish the appropriate inflammatory model based on the expression of pro-inflammatory mediators and tight junction proteins using Western blotting. After selecting the concentrations of EGCG and LPS based on cell viability and inflammatory responses, the effect of EGCG (10, 20 and 50 µmol/L) in LPS-challenged (10 µg/mL) BREC on the transcription of inflammatory cytokines and chemokines was evaluated. Activation of the NF-κB pathway targeting TLR4 was investigated using quantitative real-time (qRT)-PCR and Western blotting.

The first experimental treatments were: CON (control; no EGCG pre-treatment, no LPS induction), LPS (no EGCG pre-treatment, 10 µg/mL LPS induction), E10 + LPS (10 µmol/L EGCG pre-treatment, 10 µg/mL LPS induction), E20 + LPS (20 µmol/L EGCG pre-treatment, 10 µg/mL LPS induction) and E50 + LPS (50 µmol/L EGCG pre-treatment, 10 µg/mL LPS induction). Pre-treatment with EGCG and challenge with LPS each lasted 6 h at 37 °C. Western blotting, qRT-PCR and immunofluorescence were used to evaluate the effect of EGCG (50 µmol/L) on autophagy activation and the NLRP3 inflammasome-mediated signaling pathway in LPS-challenged (10 µg/mL) BREC.

In second experiment, we compared four groups: CON (control, no EGCG pre-treatment, no LPS induction), LPS (no EGCG pre-treatment, 10 µg/mL LPS induction), EGCG (50 µmol/L EGCG pre-treatment, no LPS induction), and EGCG + LPS (50 µmol/L EGCG pre-treatment, 10 µg/mL LPS induction). In the third experiment, autophagy was inhibited with 50 μmol/L chloroquine (CQ) for 4 h before the EGCG (50 μmol/L) pre-treatment and the LPS (10 µg/mL) induction. Four groups were compared: LPS, LPS + CQ, EGCG + LPS, and EGCG + LPS + CQ.

### Cell viability assay

Cell Counting Kit-8 (CCK-8, Solarbio, Beijing, China) was used to measure cell viability by seeding 1 × 10^4 ^cells/well of BREC into 96-well plates for 12 h before treating with different concentrations of EGCG (0, 10, 20, 50, 100, 125, 150, 200 and 300 µmol/L) for 2, 6 and 12 h at 37 °C. Subsequently, BREC were washed once with phosphate-buffered solution (PBS), 10 μL of CCK-8 was added to each well, and the BREC incubated at 37 °C for 3 h. An automated microplate reader (Molecular Devices, Shanghai, China) was used to measure the optical density (OD) at 450 nm. Cell viability was calculated as follows: (treatment group OD − blank OD)/(CON group OD − blank OD).

### RNA extraction and quantitative real-time PCR

Isolation of total RNA was performed with TRIzol reagent (Vazyme, Nanjing, China). The purity of the RNA was assessed using the A_260_/A_280_ ratio. The ratio of all samples measured was between 1.8 and 2.0, indicating a high level of purity. The integrity of the RNA was assessed using agarose gel electrophoresis. The extracted RNA sample was 1 µg and mixed with 1 µL of 5× RNA Loading Buffer before loading on a 1% agarose gel electrophoresis and run at 100–120 V for 20 min. The integrity of RNA bands was examined using a Tanon gel imaging system (Tanon, Shanghai, China). One μg of total RNA in each sample was reverse-transcribed to cDNA in a 20 μL reaction using a reverse transcription kit (RR047A, TaKaRa Biotechnology Co., Ltd.) according to the supplier's protocol. We evaluated mRNA expression levels using qRT-PCR technology with the SYBR Green QuantiTect RT-PCR Kit (RR420A; TaKaRa Biotechnology Co., Ltd.) and a 7500 Real-Time PCR System (Applied Biosystems Inc., Waltham, MA, USA). The qRT-PCR was conducted with one cycle of denaturation at 95 °C for 30 s, followed by 40 cycles with denaturation at 95 °C for 5 s and annealing extension at 60 °C for 34 s. The relative quantification values were normalized to the geometric mean of the Cq of the reference genes. Fold-changes in gene expression relative to the mean of each treatment in the control group were calculated using the 2^−ΔΔCT^ method [34]. Every component of a primer pair was located in the target gene's neighboring exons and demonstrated to anneal solely to the target sequence by Primer-BLAST software analysis. Melting curve analysis revealed that amplification with each primer pair produced a single product with efficiencies ranging from 92% to 98%. Data were analyzed by means of a relative standard curve based on serial dilutions of the pooled cDNA prepared from the cell samples. The primer pairs used in this study were designed using Primer Express software (Applied Biosystems Inc.) according to gene sequences published in GeneBank. Primers were manufactured by Sangon Biotech (Shanghai) Co., Ltd. for synthesis. Additional file 1 lists the specific primers and its efficiency values used in the quantitative PCR. Candidate reference genes tested were *ACTB* and *GAPDH*, *ACTB* was used as the internal control for data normalization as it was unaffected by physiological state.

### Biochemical analysis

BREC were pre-treated with 50 µmol/L EGCG for 6 h and then challenged with 10 µg/mL LPS for 6 h in 6-well plates (2.5 × 10^6^ cells/mL). Cell-free supernatants were subsequently centrifuged at 3,000 r/min for 20 min and the supernatant used for measuring inflammatory cytokines TNF-α (BPE92091, 4–640 ng/L), IL-6 (BPE92153, 3–480 ng/L) and IL-10 (BPE92159, 4–640 ng/L) via ELISA kits (Lengton, Shanghai, China) according to the manufacturer’s instructions. The percentage of caspase-1 activation was detected via the Caspase-1 Activity Assay Kit (Beyotime, Shanghai, China) according to manufacturer’s instructions. In brief, cells were harvested at room temperature, and the protein concentration was determined via the Bradford method. A calibration curve was constructed to determine caspase-1 activity.

### Western blotting

The Bicinchoninic acid (BCA) kit (Applygen, Beijing, China) was used for determination of protein concentration. Sodium dodecyl sulfate–polyacrylamide gel electrophoresis separation was performed with 30 µg of sample protein per lane and then proteins were transferred to polyvinylidene fluoride (PVDF) membrane (Pall, Shanghai, China) and blocked with 5% BSA. The blocked membranes were hybridized overnight at 4 °C with antibodies against β-actin (1:100,000; 66009-1-lg; Proteintech, Wuhan, China), GAPDH (1:50,000; 60004-1-lg; Proteintech), p-NF-κBp65 (1:1,000; bs-0982R; Bioss, Beijing, China), NF-κBp65 (1:1,000; 10745-1-AP; Proteintech), p-IκBα (1:1,000; MA5-16161; Invitrogen, Beijing, China), IκBα (1:10,000; 10268-1-AP; Proteintech), Occludin (1:10,000; 27260-1-AP; Proteintech) and ZO-1 (1:10,000; 21773-1-AP; Proteintech), P62(1:1,000; 66184-1-lg; Proteintech), LC3 (1:1,000; 14600-1-AP; Proteintech), NLRP3 (1:10,000; 68102-1-Ig; Proteintech) and caspase-1 (1:1,000; ab179515; Abcam, Cambridge, MA, USA). Then, the membranes were washed and incubated with horse radish peroxidase-conjugated secondary antibody (1:5,000 dilutions in TBST) at room temperature for 45 min. The final blots were developed using enhanced chemiluminescence solution (Abbkine, Wuhan, China) in a Western blotting detection system (Tanon, Shanghai, China). Image J software (National Institutes of Health, BetheSEMa, MD, USA) was used to measure band intensities.

### Immunofluorescence staining

BREC were cultured on a unique detachable chamber of a sterile Nunc Lab-Tek chamber slide system prior to staining. After adhesion, BREC were pre-treated with 50 μmol/L EGCG for 6 h, followed by incubation with 10 μg/mL LPS for 6 h. Then, BREC were fixed with 4% paraformaldehyde for 20 min. After three PBS washes, 5 min each time, the slides were permeabilized for 5 min at 4 °C with 0.2% Triton X-100 in PBS. BREC were blocked for 1 h in 5% goat serum at 4 °C, and incubated with LC3 (1:500; 14600-1-AP; Proteintech), Cytokeratin 18 (1:1,000; 10830-1-AP; Proteintech) overnight at 4 °C. BREC were then washed with PBS, incubated with CoraLite488-conjugated AffiniPure goat anti-rabbit IgG (H + L) in the dark for 1 h, and stained with 500 μL DAPI for 10 min. Immunofluorescence was visualized under a confocal laser-scanning microscope (Leica, Germany). The LC3 average fluorescence intensity were analyzed using Image J software (Mean gray value = Integrated Density/Area).

### Statistical analysis

All experiments contained at least three biological replicates. Experimental data between groups were analyzed using GraphPad Prism 8.0 (GraphPad Prism Inc., La Jolla, CA, USA) or SPSS 23.0 software (IBM Corp., Armonk, NY, USA). Data on serum samples from eight cows were normally distributed and analyzed with paired *t*-tests. All other data were normally distributed and analyzed using one-way analysis of variance (ANOVA) followed by Duncan’s multiple range test. Data are expressed as mean ± standard error of the mean. Different lowercase letters (a-f) on the bar chart indicate significant differences. Effects were deemed significant when *P* < 0.05.

## Results

### Ruminal epithelium histology and blood composition in cows with SARA

**Fig. 1:**
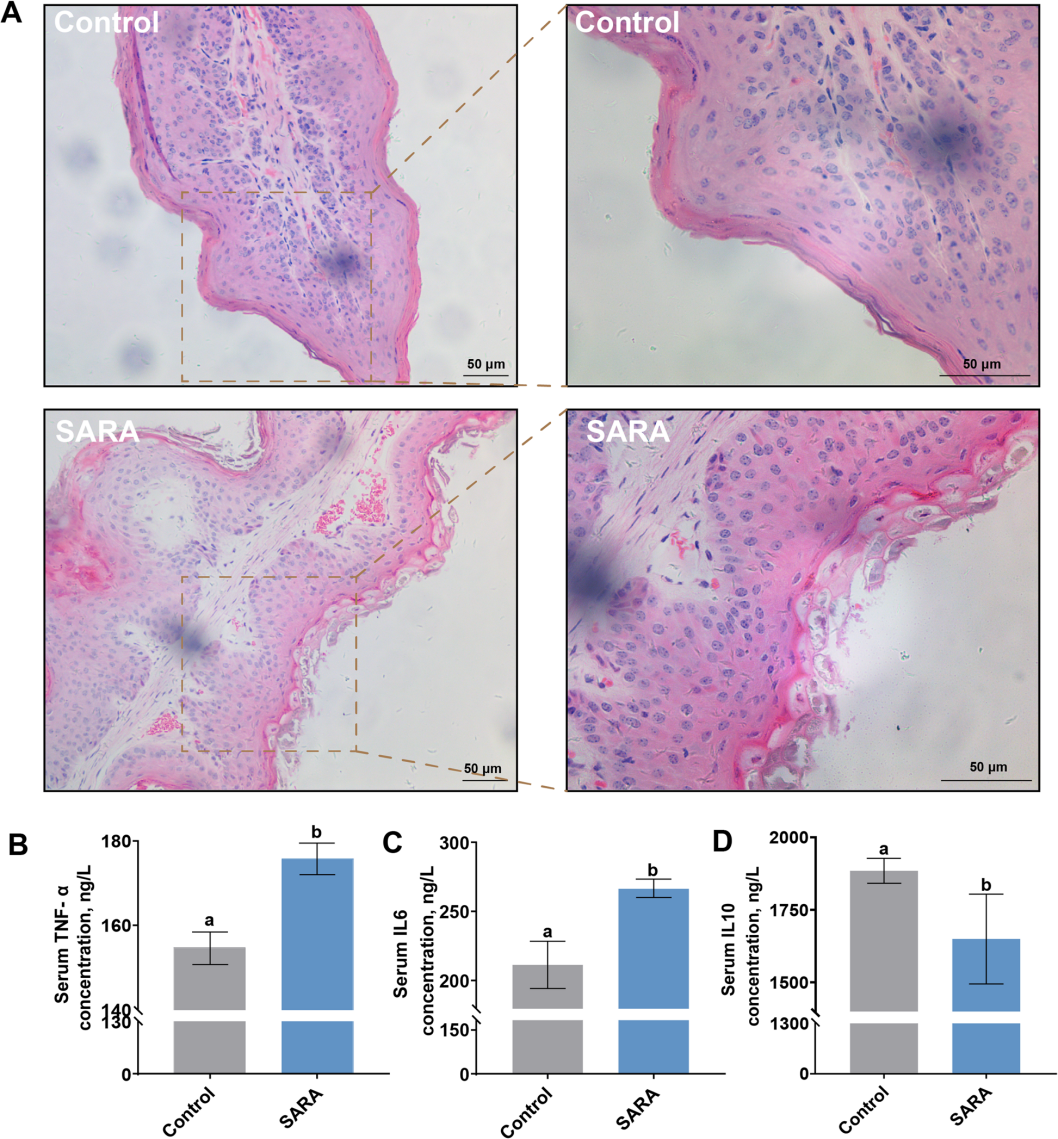
Ruminal epithelial tissue morphology and serum inflammation biomarkers. **A** H&E of ruminal epithelial tissue. Scale bar = 50 µm. **B–D** Serum proinflammatory and anti-inflammatory cytokine in dairy cows (*n* = 4). ^a,b^Values without the same letters indicate a significant difference (*P* < 0.05)

Cows with SARA had extensive shedding of the ruminal epithelial papillary stratum corneum and cell death (Fig. 1A). These cows also experienced decreased serum concentrations of the anti-inflammatory cytokine IL-10 along with increased concentrations of the proinflammatory cytokines TNF-α and IL-6 further confirming the injury of epithelial tissue and systemic inflammatory response in cows with SARA (Fig. 1B–D).

### Effects of different concentrations of EGCG on bovine ruminal epithelial cell viability

**Fig. 2:**
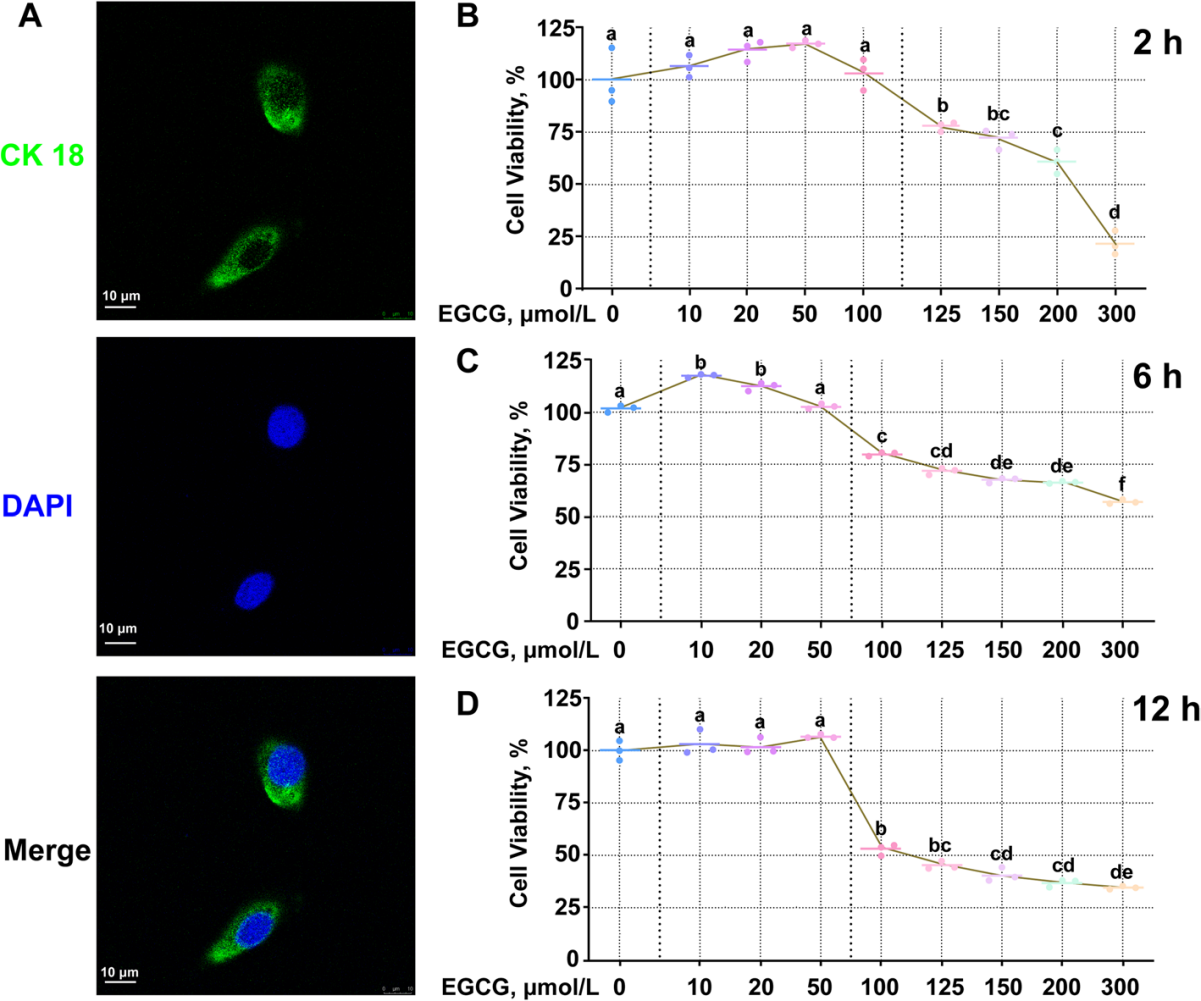
Identification of bovine ruminal epithelial cells (BREC). **A** Green light denotes Cytokeratin 18 and blue light the nucleus. Scale bar = 10 µm; Cell viability of BREC in response to epigallocatechin-3-gallate (EGCG). **B–D** BREC were treated with various concentrations of EGCG for 2, 6 and 12 h, the first dotted line distinguished the control group from the dosed group and the second dotted line represented a concentration at which the drug significantly reduced cell viability in each graph. ^a–f^Values without the same letters indicate a significant difference (*P* < 0.05)

Verification of the epithelial nature of the BREC is illustrated in Fig. 2A. Cell proliferation was not affected by EGCG concentrations equal to or below 100 µmol/L, but decreased when above 125 µmol/L EGCG for 2 h (*P* < 0.05, Fig. 2B). Treatment with 10 or 20 µmol/L EGCG for 6 h increased cell proliferation (*P* < 0.05), but for EGCG concentrations equal to or above 100 µmol/L there was a continuous and significant decrease in cell proliferation (*P* < 0.05, Fig. 2C). Cell proliferation was significantly reduced (*P* < 0.05) when BREC were incubated with EGCG concentrations great than or equal to 100 µmol/L for 12 h (Fig. 2D). Based on these findings, 10, 20 and 50 µmol/L EGCG for 6 h were chosen for subsequent studies.

### Establishment of optimal inflammatory conditions with LPS

**Fig. 3:**
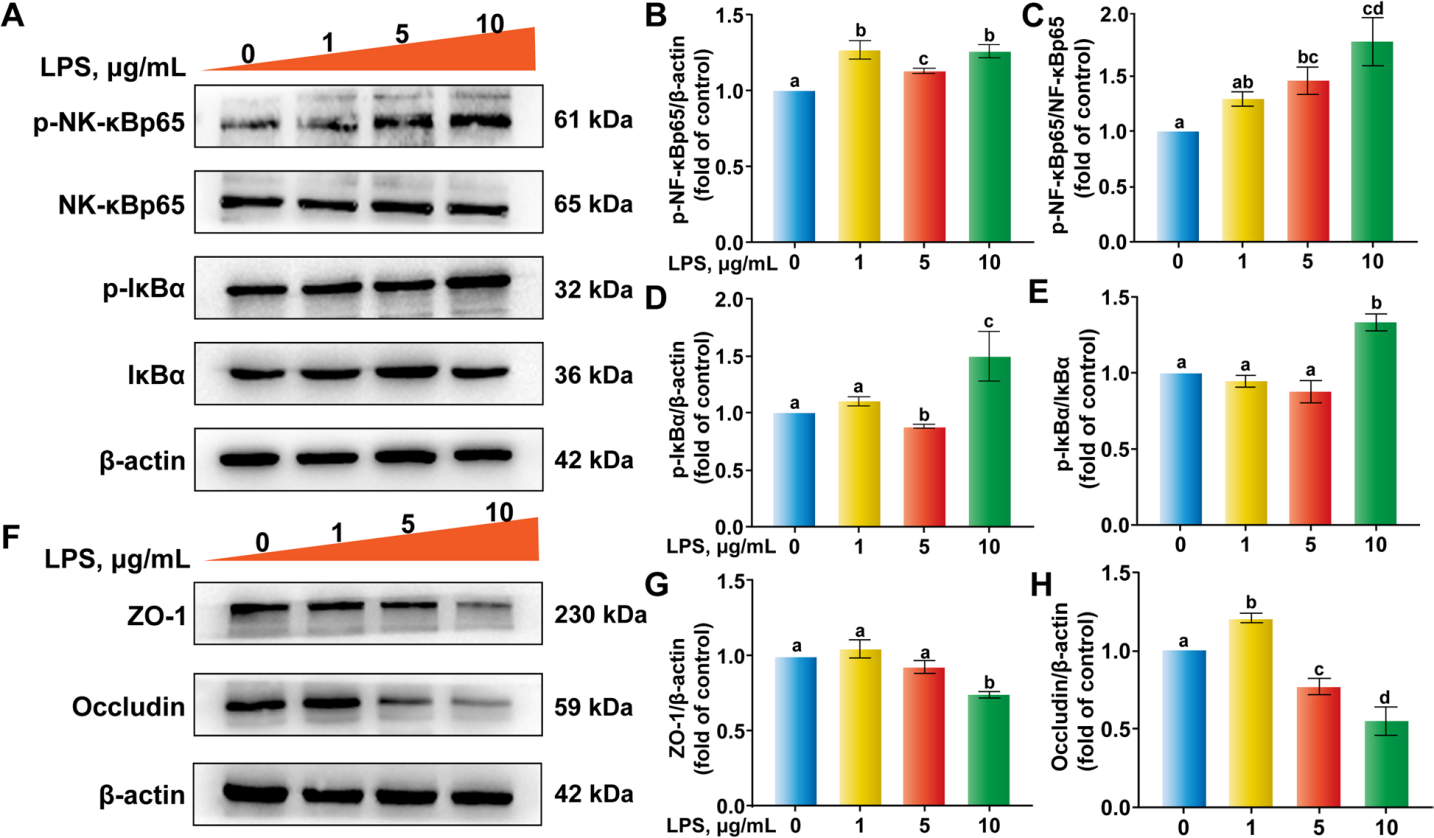
Pro-inflammatory effect and tight junction protein abundance in response to different concentrations of lipopolysaccharide (LPS) in bovine ruminal epithelial cells. Cells were treated with various concentrations of LPS for 6 h. **A–H** Western blot analysis of the abundance of phosphorylated-nuclear factor kappa-B (p-NF-κB) p65, NF-κBp65, p-IκBα, IκBα, ZO-1 and Occludin. ^a–d^Values without the same letters indicate a significant difference (*P* < 0.05)

As shown in Fig. 3A–E, increasing LPS concentrations increased phosphorylation of NF-κBp65 and IκBα, with the effect of 10 µg/mL LPS for 6 h being the most significant. Apart from the activation of the NF-κB pathway, the expression of ZO-1 and Occludin proteins also decreased significantly (Fig. 3F–H). Thus, 10 µg/mL LPS treatment for 6 h was chosen as the best model for inducing inflammation in subsequent experiments.

### Effect of EGCG on production of inflammatory cytokines and chemokines in LPS-challenged BREC

**Fig. 4:**
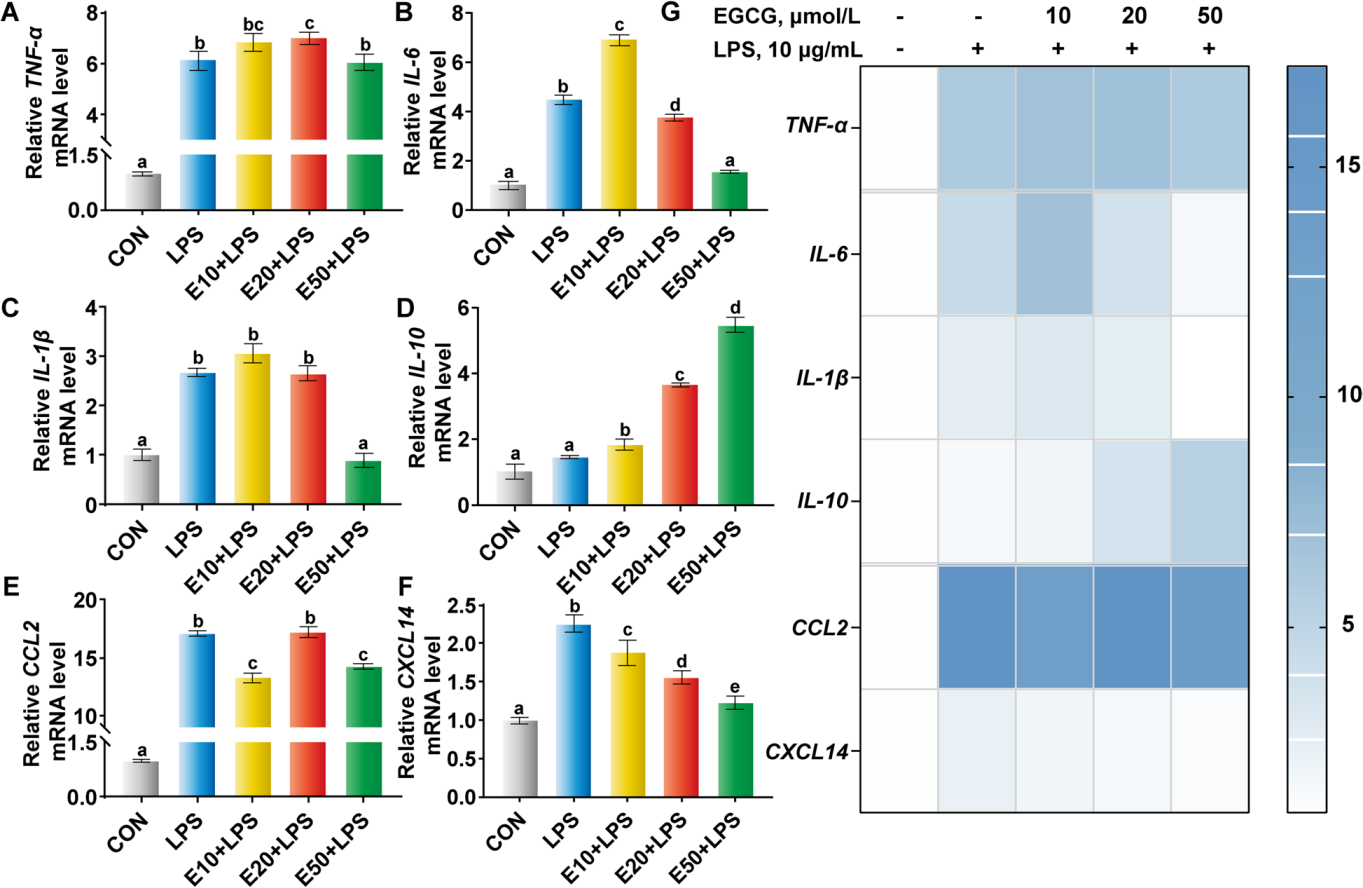
Effects of different concentrations of epigallocatechin-3-gallate (EGCG) on the production of inflammatory mediators in lipopolysaccharide (LPS)-challenged bovine ruminal epithelial cells. Cells were pre-treated with different concentrations of EGCG (10, 20, or 50 µmol/L) for 6 h before being exposed to 10 µg/mL LPS for 6 h in 6-well plates (2.5 × 10^6^ cells/mL). **A–F** The mRNA abundance of tumor necrosis factor (*TNF*)-*α*, *IL-6*, *IL-1β*, *IL-10*, C–C motif chemokine ligand 2 (*CCL2*) and C-X-C motif chemokine ligand 14 (*CXCL14*). **G** Heat map analysis of the mRNA abundance data. ^a–d^Values without the same letters indicate a significant difference (*P* < 0.05)

LPS challenge significantly upregulated the transcription of pro-inflammatory cytokines, including TNF-α, IL-6, IL-1β (Fig. 4A–C), and the chemokines C–C motif chemokine ligand 2 (CCL2) and C-X-C motif chemokine ligand 14 (CXCL14) (Fig. 4E and F). In contrast, pre-treatment with EGCG for 6 h at high concentrations (50 µmol/L) significantly inhibited the transcription of pro-inflammatory cytokines and up-regulated anti-inflammatory cytokines IL-10 that were induced by LPS (Fig. 4G).

### EGCG represses the activation of the NF-κB pathway targeting TLR4 in LPS-challenged BREC

**Fig. 5:**
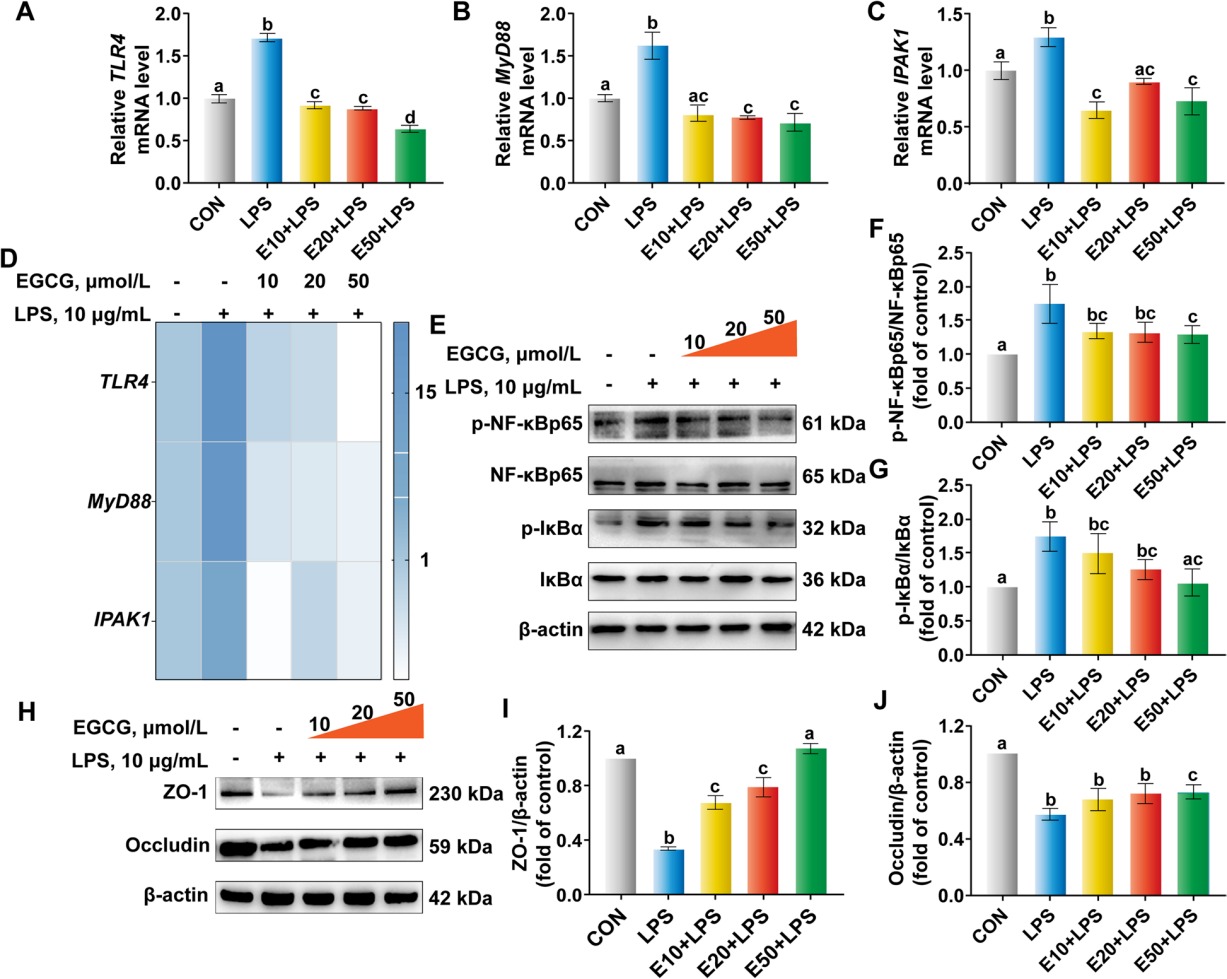
Effects of different concentrations of epigallocatechin-3-gallate (EGCG) on the nuclear factor kappa-B (NF-κB) pathway targeting Toll-like receptor 4 (TLR4). Cells were preprocessed with different concentrations of EGCG (10, 20, or 50 µmol/L) for 6 h and then added 10 µg/mL lipopolysaccharide (LPS) for 6 h in 6-well plates (2.5 × 10^6^ cells/mL). **A–C** The mRNA abundance of *TLR4*, Myeloid differentiation primary response protein 88 (*MyD88*) and Interleukin 1 receptor associated kinase 1 (*IPAK1*). **D** Heat map analysis of the mRNA abundance. **E–J** Western blot analysis of the abundance of phosphorylated-nuclear factor kappa-B (p-NF-κB p65), NF-κBp65, p-IκBα, IκBα, ZO-1 and Occludin. ^a–d^Values without the same letters indicate a significant difference (*P* < 0.05)

As evidenced in Fig. 5A–D, LPS exposure significantly upregulated (*P* < 0.05) the mRNA abundance of *TLR4*, *MyD88* and Interleukin 1 receptor associated kinase 1 (*IPAK1*), but pre-treatment with EGCG downregulated these indicators compared with the LPS group (*P* < 0.05). Fifty µmol/L EGCG pre-treatment reversed the upregulated ratio of p-IκBα to IκBα and p-NF-κBp65 to NF-κBp65 in LPS-challenged BREC (Fig. 5E–G). In addition, compared with the LPS group, 50 µmol/L EGCG pre-treatment also restored TJ protein expression of ZO-1 and Occludin (Fig. 5H–J). These results indicated that EGCG exerted anti-inflammatory activity in LPS-challenged BREC, particularly when incubated with 50 µmol/L EGCG for 6 h.

### EGCG promotes NLRP3 inflammasome degradation via activating autophagy, a critical aggravator of LPS-challenged systemic damage

**Fig. 6:**
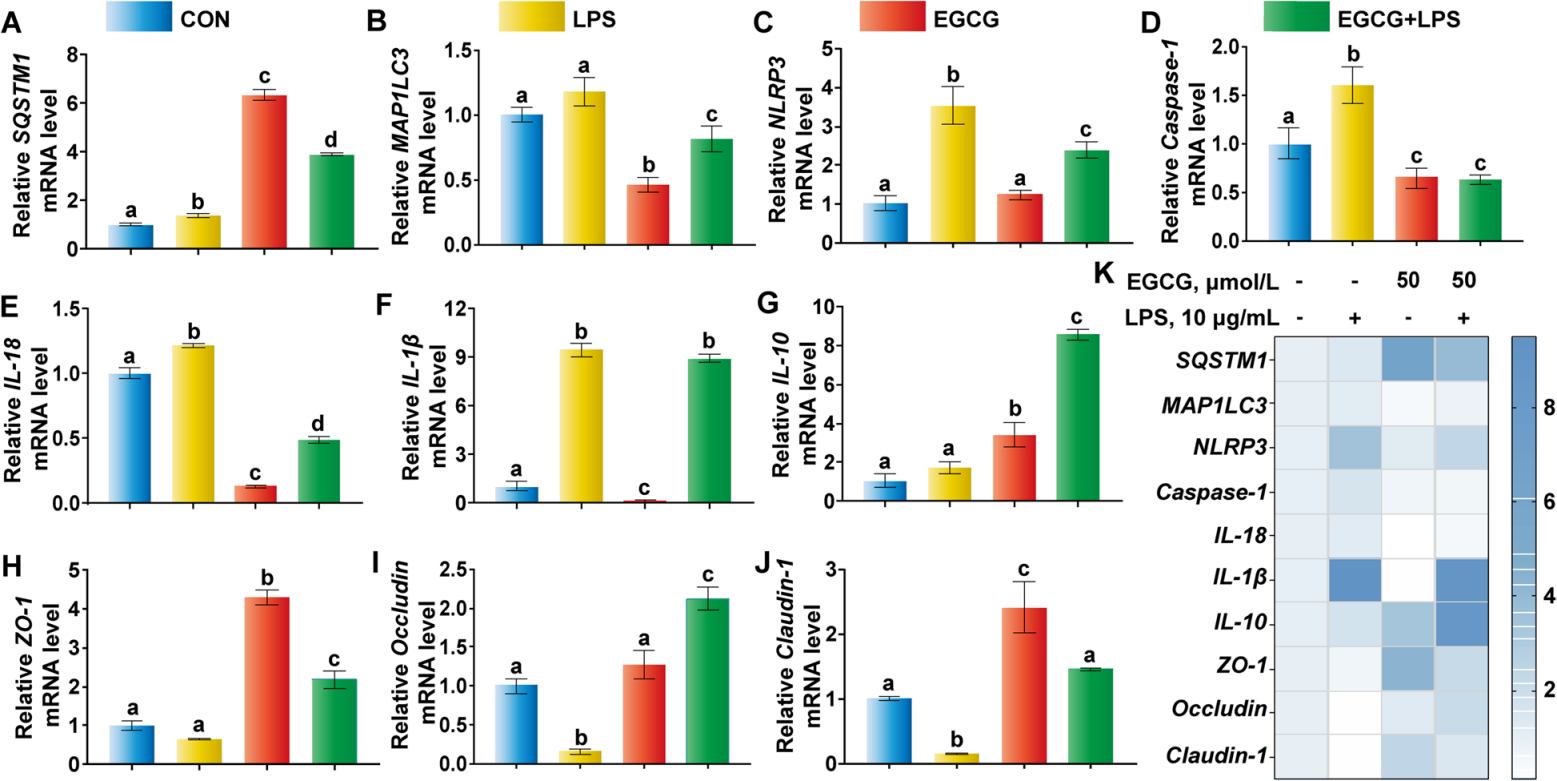
Epigallocatechin-3-gallate (EGCG) activated autophagy and NLR family pyrin domain containing 3 (NLRP3) inflammasome-mediated signaling pathway base on the mRNA expression level. BREC were pre-treated with 50 µmol/L EGCG for 6 h and then added 10 µg/mL LPS for 6 h in 6-well plates (2.5 × 10^6^ cells/mL). **A–J** The mRNA abundance of Sequestosome 1 (*SQSTM1*), Microtubule-associated protein 1 light chain 3 (*MAP1LC3*), *NLRP3*, *caspase-1*, *IL-18*, *IL-1β*, *IL-10*, *ZO-1*, Occludin, Claudin-1. **K** Heat map analysis of the mRNA abundance. ^a–d^Values without the same letters indicate a significant difference (*P* < 0.05)

Compared with the LPS group, 50 µmol/L EGCG pre-treatment for 6 h upregulated SQSTM1 and down-regulated MAP1LC3 transcription (Fig. 6A and B), and significantly reduced NLRP3 inflammasome and cleaved caspase-1 expression at the mRNA abundance level (Fig. 6C and D). In addition, the mRNA abundance of *IL-18* also decreased and the TJ mRNA abundance of *ZO-1*, Occludin and Claudin-1 upregulated due to EGCG pre-treatment in LPS-challenged BREC (Fig. 6E–J).

**Fig. 7:**
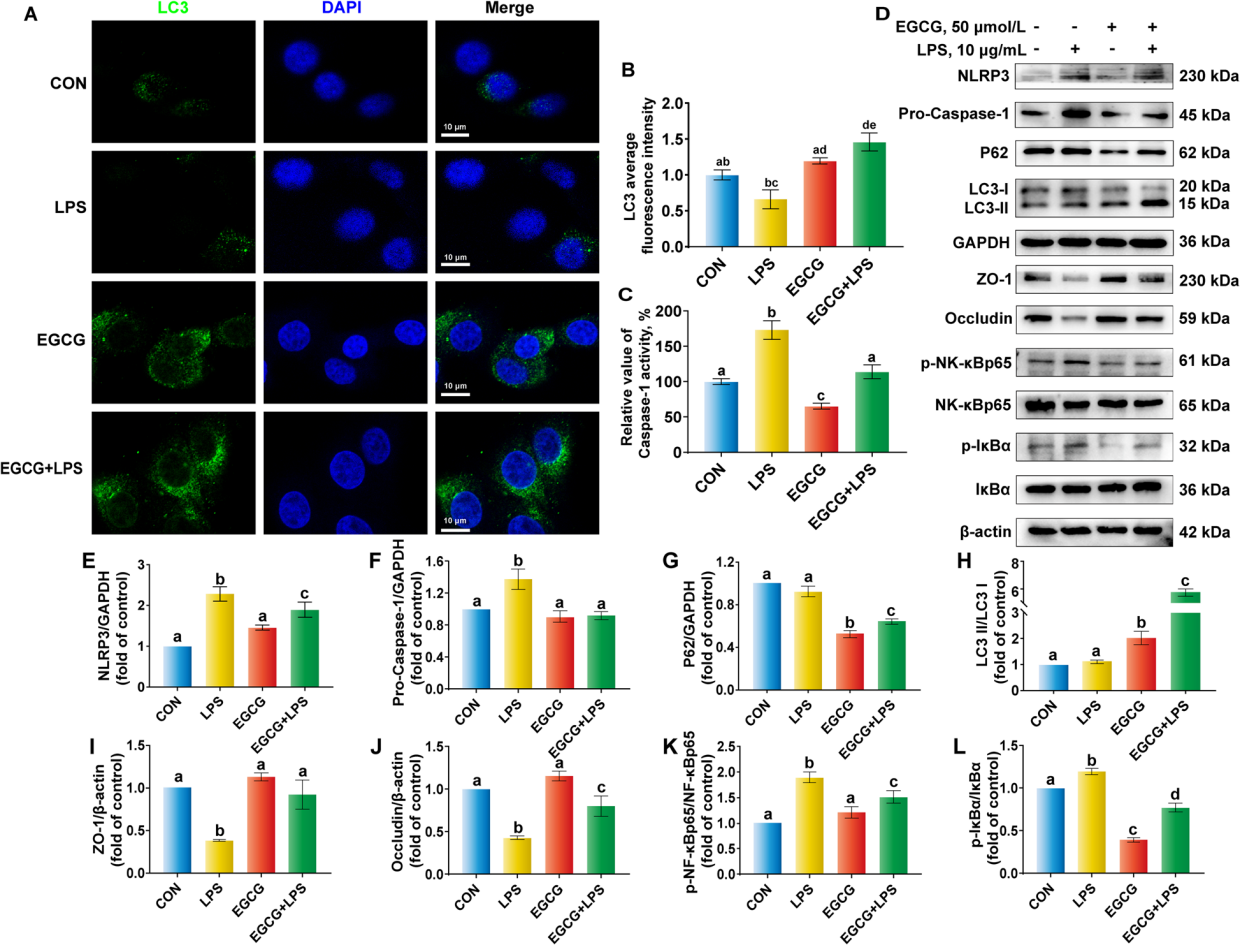
Effect of epigallocatechin-3-gallate (EGCG) on autophagy activation and NLR family pyrin domain containing 3 (NLRP3) inflammasome-mediated signaling pathway in lipopolysaccharide (LPS)-challenged bovine ruminal epithelial cells (BREC). **A** and **B** Green light represents LC3 and blue light represents nucleus. Scale bar = 10 µm; Images are representative of three independent experiments. **C** Caspase-1 activity. **D–L** Western blot analysis of the abundance of NLRP3, pro-caspase-1, p62, LC3, ZO-1, Occludin, phosphorylated-nuclear factor kappa-B (p-NF-κB) p65, NF-κBp65, p-IκBα and IκBα^a,b^Values without the same letters indicate a significant difference (*P* < 0.05)

As shown in Fig. 7A and B, the fluorescence intensity of LPS was lowest among these groups, EGCG pre-treatment activated LC3 further expression. Fifty µmol/L EGCG pre-treatment for 6 h alone had no negative impact on BREC. In contrast, compared with the control group, it decreased caspase-1 activity (Fig. 7C). Within the NLRP3 inflammasome and autophagy activation-mediated signaling pathway, it exerted the decrease in NLRP3 and pro-caspase-1 protein abundance, the increased protein abundance of LC3II and the decreased protein abundance of p62 also indicated that EGCG pre-treatment activated autophagy in BREC (Fig. 7E–H). EGCG also decreased markedly the ratio of p-NF-κBp65 to NF-κBp65 and p-IκBα to IκBα, and restored the abundance of TJ proteins under the attack of LPS (Fig. 7I–L).

**Fig. 8:**
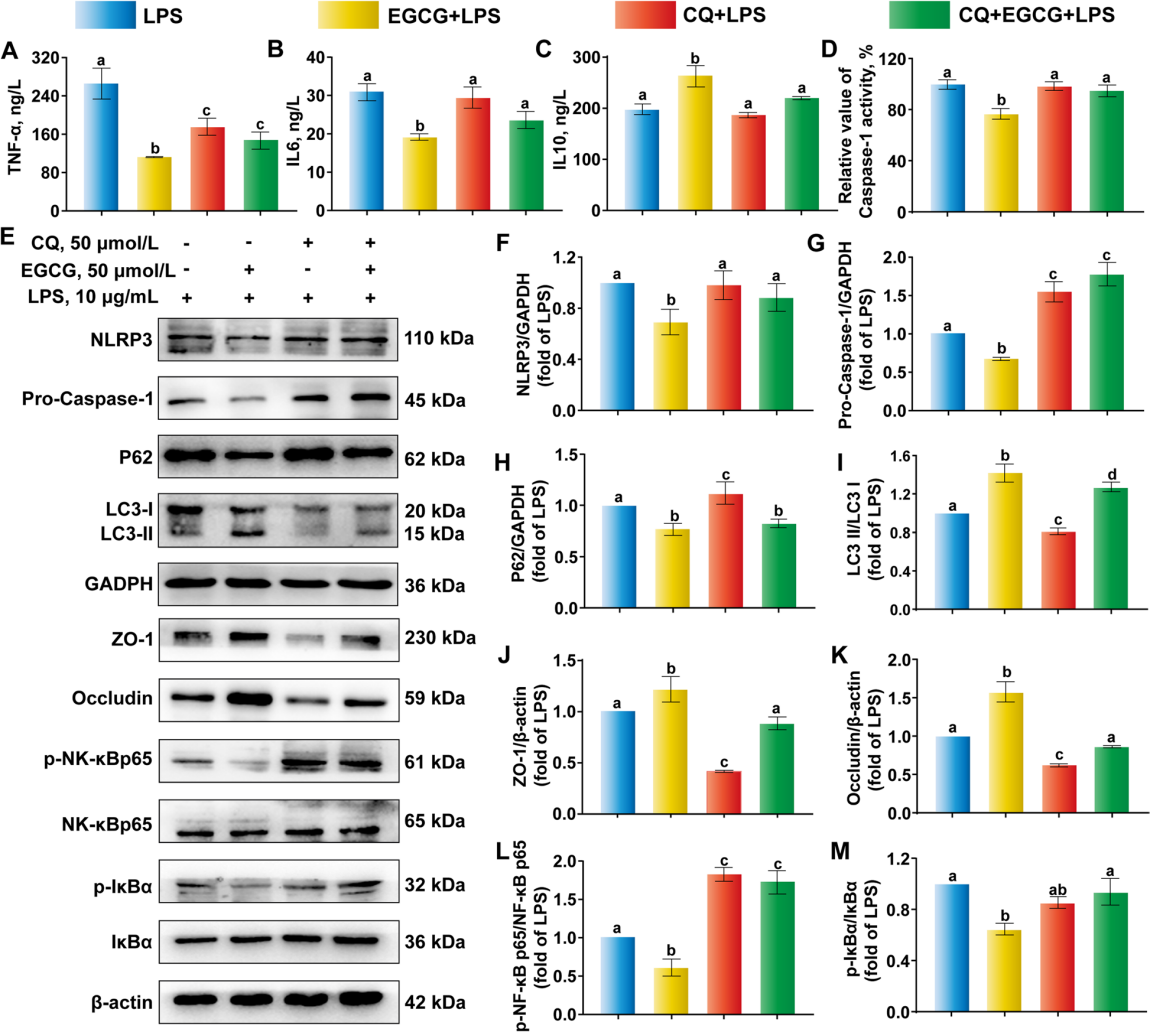
Effects of autophagy inhibition on NLR family pyrin domain containing 3 (NLRP3) inflammasome-mediated signaling pathway. Bovine ruminal epithelial cells (BREC) were pre-treated with chloroquine (50 µmol/L) for 4 h, followed by treated with epigallocatechin-3-gallate (EGCG) (50 µmol/L) for 6 h and treated with lipopolysaccharide (LPS) (10 μg/mL) for an additional 6 h. **A**–**D** The production of inflammatory cytokines of tumor necrosis factor (TNF-α), IL-6, IL-10 and the caspase-1 activity. (**E**-**M**) Western blot analysis of the expression of NLRP3, pro-caspase-1, p62, LC3, ZO-1, Occludin, phosphorylated-nuclear factor kappa-B (p-NF-κB) p65, NF-κBp65, p-IκBα and IκBα. ^a–d^Values without the same letters indicate a significant difference (*P* < 0.05)

Compared with EGCG + LPS, the anti-inflammatory cytokine IL-10 decreased significantly and proinflammatory cytokine TNF-α, IL-6 showed rising trend in the CQ + EGCG + LPS (Fig. 8A–C). The mixture of CQ + EGCG + LPS also led to a significant increase in caspase-1 activity and pro-caspase-1 protein abundance, but the NLRP3 inflammasome protein abundance was not significant (Fig. 8D, F and G). The combination with CQ remarkably reduced LC3II accumulation (Fig. 8I). Compared with EGCG + LPS, Within the NF-κB-related signaling pathway and for TJ protein abundance, CQ pre-treatment reversed the downregulation of protein abundance of the ratio of p-NF-κBp65 to NF-κBp65 and p-IκBα to IκBα in EGCG + LPS, and it also significantly downregulated protein abundance of ZO-1 and Occludin (Fig. 8J–M).

## Discussion

Subacute ruminal acidosis has emerged as one of the main metabolic disorders in high-producing dairy cattle affecting milk production, health and welfare. The onset and duration of SARA often leads to destruction of the ruminal epithelial barrier, in part due to inflammatory events that are often alleviated with the use of drugs or antibiotics [35]. Because polyphenols have been proven in a number of studies to control and alleviate inflammation [36–38], they could serve as alternatives to antibiotics as well. Our data further confirmed that SARA causes ruminal epithelium injury and systemic inflammation in vivo, and in vitro data revealed that EGCG protects BREC against LPS-induced inflammatory damage. The mechanism of action includes the activation of autophagy, thus, this polyphenol has the potential to be used as a therapeutic strategy for SARA-associated inflammation diseases. The cell viability assay via CCK8 also confirmed that at the dosages of LPS (10 µg/mL) and EGCG (10, 20 and 50 µmol/L) used in the present study there were no evident cytotoxic effects, thus, confirming the validity of the model to study the anti-inflammatory effects of EGCG.

The NLRP3 inflammasome is a multi-protein complex composed of the intracellular innate immune receptor NLRP3, the adaptor protein ASC, and the protease caspase-1 [39]. It recognizes DAMPs or PAMPs and recruits and activates the inflammatory protease caspase-1, and the latter cleaves the IL-1β and IL-18 precursors into their mature forms, thus, triggering an inflammatory response [40, 41]. The NLRP3 inflammasome can be activated by multiple exogenous and endogenous stimuli, including pathogenic infections caused by bacteria, fungi, and viruses, as well as LPS [42]. However, the fact that EGCG in vitro (at 50 µmol/L) significantly suppressed the release of pro-inflammatory cytokines due to LPS-induction, by downregulating transcription of the cytokines and chemokines, strongly suggested that this compound could be of practical value as a therapeutic tool.

At least in ruminants, there has been uncertainty as to the molecular targets affected by EGCG that can play an anti-inflammatory effect. As such, it was essential to outline the major mechanism whereby LPS triggered inflammation in BREC before we could elucidate if EGCG participated in such pathway. The increased phosphorylation of IκBα and NF-κBp65 along with the translocation of NF-κBp65 to the nucleus in response to LPS confirmed that this molecule works through the canonical NF-κB pathway [43]. Thus, the negative effect of EGCG on the mRNA abundance of TLR4 signaling components, phosphorylation of IκBα, NF-κBp65, NLRP3 and caspase-1 protein abundance confirmed that EGCG elicit an anti-inflammatory response by preventing activation of the NF-κB pathway and NLRP3 inflammasome in ruminal epithelial cells.

Because of its potential to eliminate pathogens (xenophagy) and activate immunity while limiting excessive inflammation, autophagy can impact immunity and inflammation [44]. In non-ruminants, autophagy induced by EGCG was implicated in the prevention of various human diseases such as cancer, diabetes, and cardiovascular disease [45]. In rats, exogenous EGCG also alleviated inflammatory responses by restoring autophagic flux via downregulation of Beclin1, Autophagy-related gene 5 (Atg5), and Sequestosome 1 (SQSTM1, p62) [46]. EGCG decreased dimerization of LC3B-I, enhanced LC3B-II synthesis, and activated autophagy in hepatocellular carcinoma cells [47]. The upregulation of LC3II and downregulation of p62 protein abundance (an autophagy adaptor responsible for autolysosome degradation) in response to EGCG and LPS indicated an increased in autophagic activity [48]. Together the data provide strong evidence for a link between EGCG and enhanced autophagy activation coupled with an anti-inflammatory response in the cellular response to LPS.

Non-ruminant studies over the last decades have reported that autophagy can regulate inflammasome activity, particularly NLRP3 inflammasome activation [18, 49, 50]. Our present data further confirmed that EGCG in ruminal epithelium can also reverse NLRP3-mediated caspase-1 activation and IL-1β release by activating autophagy. This was further confirmed by the inability of exogenous EGCG to activate autophagy when the BREC incubation contained the autophagy inhibitor chloroquine, i.e., both the anti-inflammatory response was reduced (NLRP3 inflammasome formation increased) and breakdown of tight junctions could not be prevented. The latter response is particularly important in the context of proper epithelial barrier function because maintenance of an intact intestinal barrier is crucial in preventing the translocation of intestinal bacteria, toxic substances, or allergens from the gut into the bloodstream [51].

In bovine, Gram-negative ruminal bacteria are the main source of LPS in the rumen [52]. Cows diagnosed with SARA often exhibit low pH levels and elevated concentrations of free LPS in the rumen, which if not treated properly can trigger a proinflammatory response [53, 54]. When the ruminal epithelium is inflamed, a decrease in barrier function increases the chance for pathogenic substances, including LPS, to enter the blood circulation and negatively affect other organs such as the liver, udder and uterus [6, 7, 55]. The present in vitro data confirmed that LPS has a negative impact on TJ proteins such as ZO-1 and Occludin and that EGCG (10, 20 and 50 µmol/L) can reverse this negative effect. Overall, the present data indicated that EGCG can act as a protective agent against LPS-induced ruminal epithelial cell inflammation and barrier injury.

## Conclusions

Subacute ruminal acidosis (SARA) can induce ruminal epithelium damage and inflammation, which would enhance transport of inflammatory factors into the bloodstream and increase the risk of systemic inflammation. The inclusion of epigallocatechin-3-gallate (EGCG), a major catechin found in green tea, in the diet could aid in preventing these responses via inducing autophagy, preventing the NF-κB inflammatory signaling pathway and formation of NLRP3 inflammasome. Further in vivo studies should be performed to confirm these findings.

### Supplementary Information

**Additional file 1**. Primer sequences used for quantitative real-time PCR.

## Data Availability

All data generated or analyzed during this study are included in this published article. Further inquiries can be directed to the corresponding author.

## Abbreviations

ACTB: β-Actin
AMPK: Adenosine 5′-monophosphate (AMP)-activated protein kinase
AP-1: Activator protein-1
ASC: Apoptosis-associated speck-like protein containing a CARD
ATG16L1: Autophagy-related protein 16-like 1
BREC: Bovine ruminal epithelial cells
CCK-8: Cell Counting Kit-8
COX: Cytochrome c oxidase
DAMPs: Danger-associated molecular patterns
EGCG: Epigallocatechin-3-gallate
GAPDH: Glyceraldehyde-3-phosphate dehydrogenase
IFN-β: Interferon-β
LPS: Lipopolysaccharides
MyD88: Myeloid differentiation primary response protein 88
NF-κB: Nuclear factor kappa-B
NLRP3: NLR family pyrin domain containing 3
NO: Nitric oxide
PAMPs: Pathogen-associated molecular patterns
ROS: Reactive oxygen species
SARA: Subacute ruminal acidosis
TJ: Tight junction
TLR4: Toll-like receptor 4
TNF: Tumor necrosis factor
