# Supplementary material for: Epigallocatechin-3-gallate protects bovine ruminal epithelial cells against lipopolysaccharide-induced inflammatory damage by activating autophagy
Source: J Anim Sci Biotechnol. 2024 Aug 9;15:109. doi: 10.1186/s40104-024-01066-9 (PMC11311925; doi:10.1186/s40104-024-01066-9)
Supplement: Supplementary file 1 — Additional file 1. Primer sequences used for quantitative real-time PCR. [file 40104_2024_1066_MOESM1_ESM.docx]

**Additional file 1** Primer sequences used for quantitative real-time PCR

| **Gene^1^** | **Primer sequence (5´→3´)^2^** | **Efficiency, %** | **Product size, bp** | **Accession number** |
| --- | --- | --- | --- | --- |
| *TNF-α* | F: GCCCTCTGGTTCAGACACTC  R: AGATGAGGTAAAGCCCGTCA | 95 | 192 | NM_173966.3 |
| *IL-6*  *IL-1β*  *ZO-1*  *CLDN1* | F: TCCAGCCACAAACACTGACC  R: ATAGCTCTCAGGCTGAACTGC  F: ACCGTACCTGAACCCATCAAC  R: TCCATCTCCCATGGAACCGA  F: TCTGCAGCAATAAAGCAGCATTTC  R: TTAGGGCACAGCATCGTATCACA  F: CGTGCCTTGATGGTGAT  R: CTGTGCCTCGTCGTCTT | 97  97  95  96 | 123  132  187  102 | NM_173923.2  NM 174093.1  XM_010817146.1    NM_001001854.2 |
| *OCLN*  *TLR4*  *MyD88*  *IRAK1*  *CCL2*  *CXCL14*  *SQSTM1*  MAP1LC3  IL-18  *IL-10*  *NLRP3*  *CASP1*  *ACTB*  *GAPDH* | F: GAACGAGAAGCGACTGTATC  R: CACTGCTGCTGTAATGAGG  F: GACCCTTGCGTACAGGTTGT  R: GGTCCAGCATCTTGGTTGAT  F: TCATTGAGAAGAGGTGCCGT  R: TGGCTTGTACTTGATGGGGAT  F: CCTCAGCGACTGGACATCCT  R: GGACGTTGGAACTCTTGACATCT  F: GCTCGCTCAGCCAGATGCAA  R: GGACACTTGCTGCTGGTGACTC  F: AAGCTGGAAATGAAGCCAAA  R: GTTCCAGGCGTTGTACCATT  F: GCCCTGACTACGACCTATGC  R: GGGATCTTCCGATGGACCAG  F: TAAGGAAACCGTGCTGCTGT  R: GCAGTGGTGTTTTTCCGTGT  F: GGCTGCCGTCTTCTGTAAG  R: GATCTGATTCCAGGTCTTCATCAT  F: GCCTTGTCGGAAATGATCCA  R: TCAGGCCCGTGGTTCTCA  F: CTTTCTGGACTCTGACCGGG  R: ATGCCTTCTCTTCCCCGTTG  F: CAGTGGTCCCTCCTTTTCCAG  R: TCCACCTTGTATCCCAGACCT  F: GCCCTGAGGCTCTCTTCCA  R: GCGGATGTCGACGTCACA  F: GGCGTGAACCACGAGAAGTATAA  R: CCTCCACGATGCCAAAGTG | 94  95  98  95  96  98  94  93  97  98  94  96  98  92 | 122  103  146  103  171    153  263  124  200  68  229  286  101  118 | NM_001082433.2  NM_174198.6  NM_001014382.2  NM_001040555.1  NM_174006.2  NM_001034410.2  NM 176641.1  NM_001001169.1    NM 174091.2      NM 174088.1      NM_001102219.1      XM_024975700.1      NM_173979.3      NM_001034034.2 |

^1^Primers were designed to measure the abundance of the following transcripts: tumor necrosis factor alpha (*TNF-α*); interleukin-6 (*IL-6*); interleukin1β (*IL-1β*); zonula occludens 1 (*ZO-1*); claudin-1 (*CLDN1*); occludin (*OCLN*); toll-like receptor 4 (*TLR4*); myeloid differentiation primary response protein 88 (*MyD88*); interleukin 1 receptor associated kinase 1 (*IRAK1*); c-c motif chemokine ligand 2 (*CCL2*); c-x-c motif chemokine ligand 14 (*CXCL14*); sequestosome 1 (*SQSTM1*); microtubule associated protein 1 light chain 3 (*MAP1LC3*); interleukin18 (*IL-18*); interleukin10 (*IL-10*); NLR family pyrin domain containing 3 inflammasome (*NLRP3*); caspase-1 (*CASP1*); β-actin (*ACTB*); glyceraldehyde-3-phosphate dehydrogenase (*GAPDH*)

^2^F, forward; R, reverse
